# Association of perinatal characteristics with biomarkers of stress and inflammation in young adults: An exploratory study

**DOI:** 10.1016/j.cpnec.2024.100249

**Published:** 2024-07-03

**Authors:** Anne-Christine Plank, Janina Maschke, Stefan Mestermann, Johanna Janson-Schmitt, Sarah Sturmbauer, Anna Eichler, Nicolas Rohleder

**Affiliations:** a Department of Child and Adolescent Mental Health, Faculty of Medicine, Friedrich-Alexander-University Erlangen-Nürnberg, Erlangen, Germany; b Chair of Health Psychology, Institute of Psychology, Friedrich-Alexander-University Erlangen-Nürnberg, Erlangen, Germany

**Keywords:** Perinatal markers, Birth weight, Cortisol, Alpha-amylase, IL-6, Stress response

## Abstract

General peri- and postnatal characteristics may serve as markers linking pre- or early postnatal events to later health outcomes, which in turn are associated with altered stress- and immune system activity. Our exploratory study investigated whether A) the common perinatal measures “birth weight” and “birth mode” and B) the postnatal characteristics “breastfeeding” and “vaccination status” are associated with markers of stress systems – the hypothalamic-pituitary-adrenal (HPA) axis and autonomous nervous system (ANS) – and inflammation in healthy young adults (*n* = 68, females: 70.6 %, mean age: 24.21 years, *SD* = 4.38) exposed to psychosocial challenge, the ‘Trier Social Stress Test’ (TSST). Salivary cortisol, alpha-amylase (sAA) and plasma interleukin-6 (IL-6) were assessed before, during and after the TSST. Participants provided information on peri- and postnatal characteristics. Linear regressions were performed to determine whether peri-/postnatal variables predict basal and stress-response-related biomarker levels. Controlling for sex and sex hormone use as relevant confounders, we found a significant association between birth weight and cortisol recovery (*p* = 0.032), with higher birth weight predicting higher cortisol recovery values. There were no other significant associations between predictor and outcome variables. Our results show that, in healthy young adults of mixed gender, normal-ranged birth weight is related to the cortisol response to psychosocial stress, indicating a long-term association of this perinatal marker with HPA axis function. In contrast, birth weight was not associated with markers of the ANS stress response or inflammation in adulthood. Our results further suggest that the measures birth mode, duration of breastfeeding, and vaccination status at 4 months of age do not relate to markers of the inflammatory and stress systems in adulthood.

## Highlights

• The study examines the association between perinatal characteristics and biomarkers of stress and inflammation in adulthood.
• Salivary cortisol, sAA and IL-6 were assessed in TSST-performing healthy young adults.
• Birth weight was significantly and positively associated with cortisol recovery.
• Vaccination status, breastfeeding and birth mode were not related to adult stress and inflammation markers.

## 1 Background

The concept of developmental origins of health and disease (DOHaD) proposes that an adult's health status and disease susceptibility is conditioned by events occurring in early life, including the prenatal period, when child development is modulated by adaptive responses to intrauterine signals transduced by the mother [1]. Accordingly, there is ample evidence that adverse exposures during fetal and early postnatal life may cause long-term alterations in various psychological and physiological traits and negatively influence the offspring's development and disease risk [1,2]. Some of the earliest observational studies related to the DOHaD concept report an association between unfavorable conditions at birth, primarily low birth weight, and increased risk of cardiovascular disease (CVD) and metabolic diseases in adult life [1,3]. However, such birth outcomes are unlikely to be causally linked to later disease susceptibility, but rather indicative of adverse prenatal conditions, including maternal over- or underweight, suboptimal dietary intake, stress, smoking and alcohol consumption during pregnancy [[4], [5], [6], [7], [8]]. Prenatal alcohol exposure (PAE) and prenatal maternal stress are commonly assessed during pregnancy or soon after birth via maternal self-reports or biomarker levels. However, the implementation of these methods in retrospective studies examining long-term effects of prenatal risks is limited: the reliability of maternal self-reports is moderate, due to under-reporting, and likely to vary depending on the time elapsed between pregnancy and reporting [[9], [10], [11]]. Biomaterial (e.g. meconium) for the determination of biomarkers - which are considered to have a higher predictive value than subjective reports [10] - is not available as standard. In contrast, objective parameters recorded at birth, such as gestational age and anthropometric measures (height, length, head circumference, and weight), can typically be retrieved from birth documents at any age. Although the nature of prenatal experiences is not specified by these parameters, they have been shown to be associated with prenatal events [[12], [13], [14]], and the original observation that adverse birth conditions are related to later risk for non-communicable somatic diseases [1] has been widely confirmed and extended to risk of psychopathology, such as anxiety disorders or ADHD [[15], [16]].

Studies provide evidence that adverse prenatal conditions are associated with long-term alterations in biological systems, including the immune system and the two key stress response systems, the autonomous nervous system (ANS) and the hypothalamic-pituitary-adrenal (HPA) axis [2,[17], [18], [19]]. Fetal programming of the HPA axis, leading to altered HPA axis activity, is considered a key mechanism linking prenatal events to long-term health consequences [20]. Evidence further suggests a relationship between perinatal measures and changes in HPA axis activity in childhood, adolescence and adulthood, such as altered basal cortisol levels and cortisol reactivity to pharmacological stimuli and psychosocial stress [[21], [22]]. Moreover, perinatal markers have been associated with cardiovascular reactivity in children, adolescents and adults [22], which is a correlate of the ANS. The ANS, in turn, mediates the acute stress response and has been linked to later disease risk [22]. The latter also applies to the immune system, especially the systemic level of pro-inflammatory markers [23] such as C-reactive protein (CRP) and interleukin-6 (IL-6). Interestingly, acute psychosocial stress provokes a transient rise in IL-6 levels [[24], [25]]. The extent of this rise has been associated with early life stress and major depressive disorder in adulthood [26,27]. The relationship between perinatal and inflammatory markers has been assessed in several studies, which demonstrated an inverse association between CRP levels and birth weight in adults [28]. Collectively, current knowledge supports the concept of fetal programming of physiological systems, linking prenatal events, birth outcomes and disease risk later in life.

Aside from the prenatal period, the DOHaD concept refers to peri- and early postnatal life events, including the process of birth itself, as well as the early and the later neonatal environment. Adults can usually self-report on peri- and postnatal characteristics that might be associated with their current health status: the delivery mode (vaginal or cesarean), whether and how long they were breastfed, and the number and timing of vaccines received as infant. In particular, these characteristics are known to influence the development of the immune system.

Neonates are capable of actively mounting an immune response to pathogens and vaccines[29], and young infants even have a high capacity to respond to multiple vaccines. Since vaccines provide protection against a range of pathogens (and potential secondary bacterial infections), they are recognized to prevent a weakening of the immune system in the long term [29]. Additionally, infants acquire a passive, temporary immunologic protection via maternal secretory immunoglobulin A (IgA) when they are breastfed. Both the nutrition received in the first months of life and the mode of delivery configure the early microbiome, and thus may influence the developing mucosal immune system. In this regard, breast milk and vaginal delivery are considered beneficial compared to formula and cesarean section [30]. Aberrant microbiota composition in infants has been shown to be associated with a chronic pro-inflammatory state and with an increased risk of disease (e.g., asthma, allergies) in childhood and later life[30].

Overall, evidence supports the concept that quantifiable, common peri- and postnatal characteristics can serve as markers linking pre- or early postnatal events to later health outcomes, which in turn are associated with altered markers of stress-system activity and inflammation. The present study aimed to extend current knowledge by investigating whether the common measures “birth weight” and “mode of delivery” (as proxies for pre- and perinatal experiences) as well as “breastfeeding” and “number of vaccinations received as infant” (as proxies for early postnatal experiences) are associated with markers of inflammation and the stress system in a cohort of clinically healthy, female and male young adults. It is assumed that measuring the responses of biological markers to acute stress adds predictive value to assessing their basal levels, since a neuroendocrine system may appear functional at rest, but may show dysregulation under psychosocial challenge [31]. We therefore exposed our participants to the Trier Social Stress Test (TSST) stress paradigm and assessed both basal and stress-induced levels of salivary cortisol as a marker of HPA axis activity, salivary alpha-amylase (sAA) as a marker of autonomic nervous system activity, and plasma IL-6 as a marker of systemic subclinical inflammation.

## 2 Methods

### 2.1 Study design and participants

Participants were recruited from the Friedrich-Alexander-University Erlangen-Nürnberg campus by the Chair of Health Psychology to participate in a study investigating effects of coping mechanisms on HPA axis, sympathetic nervous system (SNS) and inflammatory activity [32,33]. Volunteers were eligible for the study if they met the following inclusion criteria: 1) minimum age of 18 years, 2) body mass index (BMI) between 18 and 30 kg/m^2^, 3) no previous experience with the TSST, 4) non-smoker, 5) not addicted to alcohol or illicit drugs, 6) not taking medications (e.g., anti-depressants, beta blockers, glucocorticoids) except for sex-hormonal agents, 7) absence of physical or mental disorders, 8) no major psychiatric disorders or psychiatric symptoms as measured by the German version of the Centers for Epidemiological Studies Depression Scale (CES-D) [34], termed “Allgemeine Depressionsskala” (ADS-L) [35], with scores >22 defined as exclusion criterion for participation, since scores above this cutoff indicate the presence of depressive symptomatology [35]**.** Participants (*n* = 162) underwent a laboratory visit, which comprised repeated blood and saliva sampling before and after performing the TSST (see section “procedures”). In 2021, participants were contacted again and asked for additional information on peri- and postnatal characteristics for the present study. Data was provided via online self-report by *n* = 68 participants. These did not differ significantly from the *n* = 94 non-participating subjects regarding their age (*t* = −0.474, *p* = 0.636), sex (*χ*^*2*^ = 0.929, *p* = 0.335) or BMI (*t* = −0.697, *p* = 0.487). The study was approved by the Ethics Committee of the Friedrich-Alexander-University Erlangen-Nürnberg Medical School. All participants provided written informed consent before study entry.

### 2.2 Experimental procedures

Details on the laboratory visit, including the TSST procedure and biomarker sampling, are described by Janson and Rohleder [32]. Briefly, the laboratory visit was scheduled between 2:00 p.m. and 6:00 p.m. to control for circadian variations in biomarker levels. Participants were also instructed to refrain from exercising for 24 h prior to the visit, and from brushing teeth, eating or drinking anything except water for 1 h before the visit. Female participants who did not use oral contraceptives performed the test in the luteal phase of their menstrual cycle. After initial placement of a peripheral venous catheter for blood draws in a subsample of participants (*n* = 45 out of *n* = 68) and a 45-min resting period, a first sample of saliva and blood was collected immediately before introduction to the TSST (T0). Additional saliva samples were collected at +1 min (T1), +10 min (T2), +20 min (T3), +30 min (T4), +45 min (T5) and +60 min (T6), additional blood samples at +30 min (T4) and +120 min (T7) after the TSST.

## 3 Measures

### 3.1 Biomarkers

#### 3.1.1 Salivary cortisol and alpha-amylase (sAA)

To investigate the baseline status and reactivity of the stress system, salivary cortisol and alpha-amylase were assessed as markers for the HPA axis and the SNS, respectively.

Saliva samples were collected via Salivettes (Sarstedt, Nümbrecht, Germany) according to the manufacturer's instructions at T0-T6 and were stored for later assessment at −20 °C. For analysis, samples were thawed at room temperature and centrifuged at 2000×*g* and 20 °C for 5 min. Cortisol levels were assessed in duplicate using a chemiluminescence immunoassay (CLIA, IBL International, Hamburg, Germany). Concentrations of sAA were determined with an in-house enzyme kinetic assay using reagents from DiaSys Diagnostic Systems GmbH (Holzheim, Germany), as previously described [36]. Intra- and inter-assay coefficients of variation (CVs) were below 10 %.

#### 3.1.2 IL-6 plasma levels

To quantify low-grade systemic inflammation, levels of IL-6 were assessed in plasma samples. Blood was collected into EDTA-coated monovettes at T0, T4 and T7 and centrifuged (1000 *× g*, 15 min), plasma samples were aliquoted and stored at −80 °C until analysis. Concentrations of IL-6 were determined using a commercial high-sensitivity ELISA kit (Quantikine HS; R&D Systems, Minneapolis, MN, USA) according to the manufacturer's instructions. The assay had a lower limit of detection of 0.09 pg/ml and both the intra- and inter-assay CV was below 10 %.

### 3.2 Perinatal data

Participants were requested to retrieve information on the following peri- and postnatal measurements from their mother's maternity pass or their pediatrician's examination booklet: weeks of completed pregnancy (gestational age), birth weight (in grams), length at birth (in centimeters), head circumference (in centimeters), and birth mode (spontaneous vs. caesarean (C-) section). They were also asked to provide information on their breastfeeding history (no/yes, if yes: duration in months) and their immunization status as an infant. The latter was assessed from digital copies of their vaccination certificates. Recommended standard vaccinations for infants in Germany are [37]: poliomyelitis, diphtheria, tetanus, pertussis, *Haemophilus influenzae* type B, pneumococci, hepatitis B, rotavirus (first vaccine dose at 2 months of age) and measles, mumps, rubella, varicella (first vaccine dose at 11 months of age). A dichotomous variable was formed by classifying the number of received vaccinations between birth and 4 months of age (group A: zero to two vaccinations, group B: three to seven vaccinations).

### 3.3 Potential confounders

The following variables were considered as potential confounders and assessed at the time of the laboratory visits: sex, age, body mass index [BMI, (kg/m^2^)], sex hormone use (e.g., oral contraceptives), perceived chronic stress over the past four weeks [assessed via the German version of the 10-item Perceived Stress Scale (PSS) [38] and measured as overall sum scores [32] and self-reported depressive symptoms [assessed with the 20-item ADS-L [35], the German version of the CES-D [34] and measured as overall sum scores [].

## 4 Data analysis

Statistical analyses were performed using IBM® SPSS® Statistics, Version 28 (IBM Corp., Armonk, NY, USA). Shapiro-Wilk tests were applied to check for normal distribution of outcome variables (cortisol, sAA and IL-6 values); in order to reduce skewness, all values were log-transformed and subsequent data analyses were conducted with these transformed data. Based on the repeated measurements of cortisol, sAA and IL-6 levels, the following outcome variables were defined: *baseline*: level measured at T0; *reactivity*: peak value minus baseline value; *recovery* (cortisol, sAA): peak value minus value obtained at T6 (cortisol)/T5 (sAA). For each analysis, only participants who provided valid values for all required variables were included. Missing data were due to exclusion of outliers (values deviating more than three standard deviations (SD) from the mean), incomplete information provided, lack or insufficient quality of the saliva or blood sample at the respective sampling time, or non-responder (reactivity values ≤ 0). Accordingly, sample sizes vary among analyses, and different sample sizes are indicated in the notes for the respective tables. To check for potential collinearity of predicting variables, associations between perinatal measures were evaluated via *t*-tests and Pearson correlations. To test for the assumption of homogeneity of variance, Levene's test was used and if the assumption was not met, degrees of freedom (df) were adjusted. Associations between gestational age, birth weight, head circumference and length at birth were found to be significant (*r* > 0.48, *p* < 0.001), and birth weight was selected as predicting variable. No collinearity was found regarding the other predicting variables, i.e., birth mode, breastfeeding and vaccination status. Associations between potential confounders and outcome variables were tested via Pearson correlations (age, BMI, PSS, ADS-L) or *t*-tests (sex, sex hormone use). Relevant confounders were tested for associations with predicting variables, confirming non-collinearity (*r* < 0.30), and included in the first block of hierarchical multiple linear regression analyses, which were performed to assess the influence of the perinatal predictors (*model 1*, second block: birth weight and birth mode; *model 2*, second block: breastfeeding and vaccination status) on cortisol, sAA and IL-6 baseline levels and stress reactivity measures. For interpretation of multiple regressions, *R*^*2*^, standardized beta (*β*) and *p*-values are reported. Effect sizes were classified according to Cohen [39] into small (0.10 ≤ |*β* or *r*| < 0.30), medium (0.30 ≤ |*β* or *r*| < 0.50) or large (|*β* or *r*| ≥ 0.50). Due to the exploratory nature of our study, we did not correct for multiple testing. For all analyses, the probability of error was 5 % as the level of significance was defined as *p* < 0.05 (two-tailed); *p* < 0.10 was interpreted as a trend effect.

## 5 Results

### 5.1 Sample characteristics

Descriptive statistics of the sample's characteristics are presented in Table 1. The sample comprised 48 (70.6 %) females and 20 (29.4 %) males.

**Table 1:** Sample characteristics.

peri-/postnatal measures	*n*	*mean (SD)*	*min*	*max*
gestational age (weeks)	63	39.59 (1.38)	35	42
birth weight (gram)	62	3434.95 (426.27)	2500	4400
birth weight women^a)^	44	3382.20 (447.84)	2500	4400
birth weight men^a)^	18	3563.89 (346.26)	2730	4250
length (cm)	61	51.49 (2.57)	41	57
head circumference (cm)	60	35.21 (2.29)	32	49
breastfeeding (months)	60	6.39 (4.38)	0	18
birth mode	63	spontaneous:	C-section: n = 13 (20.6 %)	
		n = 50 (79.4 %)		
vaccination status (at 4 months of age)	60	0-2 vaccinations: n = 27 (45 %)	3-7 vaccinations: n = 33 (55 %)	

**potential confounders**	***n***	***mean (SD)***	***min***	***max***
age	68	24.21 (4.38)	18	38
BMI	68	22.35 (2.30)	19.05	28.38
chronic stress (PSS overall sum score)	68	26.88 (6.13)	13	42
depressive symptoms (ADS-L overall sum score)	68	12.43 (5.72)	0	22
sex hormone use	68	yes:	no:	
		*n* = 20 (females)	*n* = 48	

**outcome measures**	**females**		**males**	
	***n***	***mean (SD)***^***b)***^	***n***	***mean (SD)***^***b)***^
cortisol baseline	46	0.70 (0.28)	20	0.78 (0.28)
cortisol reactivity	34	0.32 (0.24)	18	0.51 (0.24)
cortisol recovery	34	0.31 (0.17)	18	0.43 (0.13)
sAA baseline	48	1.68 (0.44)	20	1.90 (0.45)
sAA reactivity	43	0.37 (0.24)	17	0.32 (0.17)
sAA recovery	43	0.31 (0.26)	17	0.33 (0.23)
IL-6 baseline	32	−0.11 (0.29)	11	−0.20 (0.30)
IL-6 reactivity	30	0.63 (0.31)	9	0.64 (0.42)

Notes: C-section: Caesarian section; BMI: body-mass-index (kg/m^2^); PSS: Perceived Stress Scale (maximum sum score = 50); ADS-L: “Allgemeine Depressionsskala (Langform)” (maximum sum score = 80). Statistics: a) *t* (60) = −1,540, *p* = 0.129; b) lg-transformed values (baseline)/calculated from lg-transformed values (reactivity/recovery).

### 5.2 Pre-analyses

**Fig. 1:**
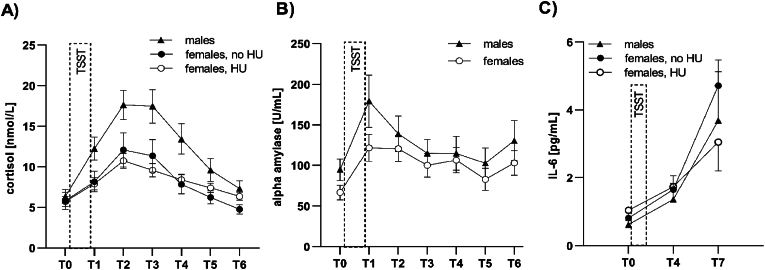
Salivary cortisol (A), salivary alpha-amylase (B) and plasma IL-6 (C) responses of female and male participants to the TSST. Data points: mean values, error bars: standard error of the mean. HU: (sex) hormone use. (A) males: *n* = 18; females, no HU: *n* = 16; females, HU: *n* = 18; (B) males: *n* = 17, females: *n* = 43; (C): males: *n* = 9; females, no HU: *n* = 20; females, HU: *n* = 10.

Fig. 1 shows the expected change of mean salivary cortisol (Fig. 1A), salivary alpha-amylase (Fig. 1B) and plasma IL-6 (Fig. 1C) levels in the context of the TSST, excluding non-responders (= reactivity values ≤ 0; cortisol: *n* = 14; sAA: *n* = 8; IL-6: *n* = 4). Participants showing a cortisol response did not differ significantly from non-responders regarding birth weight (*t*(59) = −1.105, *p* = 0.274), birth mode (*Χ*^*2*^(1) = 0.309, *p* = 0.578) breastfeeding (*t*(57) = 0.851, *p* = 0.398) and vaccination status (*Χ*^*2*^(1) = 0.011, *p* = 0.918). Non-responders could not be taken into account in reactivity analyses due to the limited size of the sample, and were therefore excluded. Raw associations between predictors and outcome variables were tested via Pearson correlations, revealing significant positive associations between birth weight and cortisol reactivity (*r* = 0.317, *p* = 0.03; n = 47), birth weight and cortisol recovery (*r* = 0.419, *p* = 0.003; *n* = 47) and vaccination status and sAA baseline (*r* = 0.259, *p* = 0.046; *n* = 60). Testing the influence of potential confounders (sex, age, BMI, sex hormone use, perceived chronic stress and depressive symptoms) on outcome variables revealed a significant effect of sex on cortisol reactivity (*t*(50) = −2.66, *p* = 0.011) and cortisol recovery (*t*(50) = −2.69, *p* = 0.010), as well as a trend-effect of sex on sAA baseline values (*t*(66) = −1.81, *p* = 0.075), with males displaying higher values in all cases (Fig. 1A and B). Sex was therefore included as covariate in cortisol and sAA regression analyses. Since females using sex hormones showed significantly lower cortisol recovery values (*t*(32) = −2.57, *p* = 0.015) and a trend to lower IL-6 reactivity (*t*(28) = −1.82, *p* = 0.079) (Fig. 1B and C), sex hormone use (yes vs. no) was taken into account as covariate in cortisol and IL-6 regression analyses for both sexes. No significant associations were found for the variables age, BMI, stress and depressive symptoms.

### 5.3 Association of birth weight and birth mode with outcome variables

Multiple regressions examining whether birth weight and birth mode predict outcome variables showed a significant fit of the model for cortisol reactivity values (*R*^*2*^ = 0.212, *p* = 0.036), with trend-effects indicating an association of sex (medium effect size) and a positive relationship of birth weight (small effect size) with cortisol reactivity (Table 2). Moreover, a significant fit was revealed for the model predicting cortisol recovery values (*R*^*2*^ = 0.348, *p* = 0.001). Birth weight was found to be a significant positive predictor, even though the effect size of sex hormone use (medium: *β* = 0.378) was larger than the effect size of birth weight (small: *β* = 0.290) (Table 2). The cortisol reactivity and cortisol recovery values entered in the model (*n* = 47) showed a high correlation (*r* = 0.509; p < 0.001).

**Table 2:** Multiple regression analyses predicting outcome variables from birth weight and birth mode.

Outcome variable	*n*	Step 1			Step 2				
		sex	sex hormone use		sex	sex hormone use	birth weight	birth mode	
		β (p)	β (p)	R^2^ (p)	β (p)	β (p)	β (p)	β (p)	R^2^ (p)
**Cortisol baseline**	60	0.136 ns	−0.007 ns	0.018 ns	0.127 ns	−0.014 ns	0.044 ns	−0.017 ns	0.020 ns
**Cortisol reactivity**^**a)**^	47	0**.381 (**0**.026)**	−0.006 ns	0**.143 (**0**.033)**	0.322 (0.072)	−0.024 ns	0.252 (0.086)	−0.091 ns	0**.212 (**0**.036)**
**Cortisol recovery**^**b)**^	47	0.174 ns	0**.376 (**0**.020)**	0**.242 (**0**.002)**	0.084 ns	0**.378 (**0**.026)**	0**.290 (**0**.032)**	−0.155 ns	0**.348 (**0**.001)**
**sAA**	62	0**.294 (**0**.020)**		0**.086 (**0**.020)**	0.307 (0.021)		−0.063 ns	0.007 ns	0.090 ns
**baseline**									
**sAA reactivity**	56	−0.131 ns		0.017 ns	−0.125 ns		−0.067 ns	−0.040 ns	0.023 ns
**sAA recovery**	56	0.020 ns		0.000 ns	0.009 ns		0.146 ns	0.113 ns	0.035 ns
**IL-6 baseline**	39		−0.051 ns	0.003 ns		−0.018 ns	−0.159 ns	0.077 ns	0.029 ns
**IL-6 reactivity**	35		0.265 ns	0.070 ns		0.372 (0.044)	−0.107 ns	−0.298 (0.089)	0.165 ns

Notes: **a)** females: *n* = 31; **b)** sex hormone use “yes”: *n* = 17; **c)** females: *n* = 44; ns: not significant.

The models examining associations of all relevant predictors with baseline values, sAA- and IL-6 stress response values were not statistically significant (Table 2).

### 5.4 Association of breastfeeding and vaccination status with outcome variables

Multiple regressions were further applied to examine whether vaccination status (at 4 months of age) and breastfeeding (duration in months) predicted outcome variables. A significant fit was found for the model predicting cortisol reactivity values (*R*^*2*^ = 0.248, *p* = 0.032), yet sex remained the only significant predictor with a large effect size (*β* = 0.508, *p* = 0.006) in the second step of the model (Table 3). A significant fit was further reported for cortisol recovery values (*R*^*2*^ = 0.321, *p* = 0.006), with a small trend-effect of breastfeeding (positive relationship) and a medium trend-effect of sex hormone use (Table 3). The cortisol reactivity and cortisol recovery values entered in this model (*n* = 41) showed a high correlation (*r* = 0.546, p < 0.001).

**Table 3:** Multiple regression analyses predicting outcome variables from breastfeeding and vaccination status.

Outcome variable	*n*	Step 1			Step 2				
		sex	sex hormone use		sex	sex hormone use	breast- feeding	vaccination status	
		*β (p)*	*β (p)*	R^2^ (p)	*β (p)*	*β (p)*	*β (p)*	*β (p)*	R^2^ (p)
**Cortisol baseline**	51	0.104 ns	−0.057 ns	0.009 ns	0.110 ns	−0.048 ns	0.131 ns	0.010 ns	0.026 ns
**Cortisol reactivity**^**a)**^	41	0**.474 (**0**.007)**	−0.013 ns	0**.219 (**0**.009)**	0**.508 (**0**.006)**	−0.041 ns	0.154 ns	0.064 ns	0**.248 (**0**.032)**
**Cortisol recovery**^**b)**^	41	0.196 ns	0**.351 (**0**.041)**	0**.232 (**0**.007)**	0.255 ns	0.300 (0.101)	0.269 (0.062)	0.114 ns	0**.321 (**0**.006)**
**sAA** **baseline**^**c)**^	53	0**.357 (**0**.009)**		0**.127 (**0**.009)**	0.**342 (**0**.012)**		−0.052 ns	0.222 (0.094)	0**.178 (**0**.022)**
**sAA reactivity**	48	−0.143 ns		0.021 ns	−0.161 ns		−0.094 ns	−0.012 ns	0.029 ns
**sAA recovery**	48	−0.036 ns		0.001 ns	−0.078 ns		−0.151 ns	0.236 ns	0.076 ns
**IL-6 baseline**	33		−0.196 ns	0.038 ns		−0.242 ns	−0.193 ns	0.088 ns	0.080 ns
**IL-6 reactivity**	30		0.288 ns	0.083 ns		0.261 ns	0.007 ns	0.100 ns	0.092 ns

Notes: **a)** females: n = 28**; b)** sex hormone use “yes”: n = 15; **c)** females: *n* = 38; ns: not significant.

The significant fit of the model for sAA baseline values (*R*^*2*^ = 0.178, *p* = 0.022) was mainly explained by the significant effect of sex with a medium effect size (*β =* 0.342, *p* = 0.012) (Table 3). Additionally, a trend to a positive association of the vaccination status with sAA baseline values was observed (Table 3).

No significant fit was reported for the models predicting cortisol baseline, sAA response and IL-6 values from breastfeeding and vaccination status (Table 3).

## 6 Discussion

There is substantial evidence that perinatal measures are associated with later somatic and psychological health outcomes, which come along with altered activity of the stress- and immune system. In the present study, we used data from a cohort of clinically healthy young adults exposed to psychosocial challenge to investigate whether the common perinatal measures “birth weight” and “mode of delivery” and the postnatal characteristics “breastfeeding” and “number of vaccinations received as infant” are associated with markers of inflammation, the stress system and its response to acute stress.

A broad body of literature suggests relations between maternal stress during pregnancy, low birth weight and both somatic and psychiatric disorders in adulthood [14,22]. Fetal programming of the HPA axis, leading to changes in HPA axis activity, is regarded as a pivotal mechanism linking prenatal events with adverse health outcomes in later life. Depending on the disorder, both a hyperactivity [e.g., cardiovascular disease [40] and a hypoactivity [e.g., posttraumatic stress disorder [41] of the HPA axis have been observed. Several studies report an association between low birth weight and alterations in HPA axis function in adulthood, such as increased basal morning cortisol levels and altered cortisol responses to pharmaceutical challenge [[42], [43], [44]].

In our study sample, we did not observe a primary association between birth weight and baseline cortisol levels, measured at mid-day before the TSST. However, after controlling for the known confounding factors sex and sex hormone use [45] and accounting for birth mode, birth weight was found to be a significant positive predictor for post-stressor cortisol recovery. Interpreting statistical trends and effect size measures, our data further indicated that birth weight was positively associated with cortisol reactivity to the TSST, which, in turn, was highly correlated with cortisol recovery values. To the best of our knowledge, this study is the first to investigate the link between birth weight and cortisol response to psychosocial challenge induced by the TSST cortisol in a young adult cohort of mixed gender. Our findings extend previous research, where not all results have been consistent. In a cohort of male adult twins [21], a higher cortisol response was observed in participants with lower birth weight, while cortisol baseline values were not associated with birth weight. The same findings were reported for 7- to 9-year-old boys by Jones and colleagues [46] – interestingly, no association between birth weight and cortisol response was found in girls of the same age group [46]. A study in young female and male adults reported on a blunted cortisol response in participants with very low birth weight (≤1500 g) [47]**.** In older male and female adults (mean age: 63 years), Kajantie et al. observed an inverse U-shaped relationship, where subjects in the lowest third of birth weights (<3125 g) showed the lowest cortisol responses [48].

It cannot be ruled out that these divergent results might be due to different analytic strategies and/or sample characteristics, such as sample size, age, gender, origin and birth weight range. However, since both hyper- and hypoactivity of the HPA axis have been associated with later health outcomes, both a positive and an inverse association between birth weight and cortisol response could support the hypothesis of a fetal programming of the HPA axis. It has to be noted that the effect size of birth weight predicting cortisol recovery in our cohort was small. This is not surprising, since, one the one hand, there are additional factors that may influence the cortisol stress response, such as different stress coping strategies [32], genetic factors, or prolonged periods of stress earlier in life, which were not captured by the PSS-10 or ADS-L. On the other hand, birth weight is also influenced by many individual factors, and the birth weight of our cohort was largely within the physiological range as participants were recruited from the general population.

These aspects may also account for the fact that we did not observe any association between birth weight and SNS reactivity, assessed by sAA levels. SAA is an established marker of SNS activity and reactivity [49], i.e., the immediate “fight or flight” stress response leading to increased heart rate, blood pressure, and epinephrine/norepinephrine release. Importantly, sAA has been shown to correlate with plasma-norepinephrine levels [49]. To our knowledge, our study is the first to explore a link between perinatal markers and SNS reactivity quantified by sAA levels. Previous studies examining blood pressure, vascular resistance, heart rate or catecholamine release have provided mixed results summarized in Ref. [22]: In 7- to 9-year-old children, a sex-dependent association was found between birth weight and autonomic stress response to the TSST was found [50,51]. In adults, two studies using a different stressor report on links between lower birth weight and higher cardiovascular reactivity in women, but not in men [52,53]. Another study applying the TSST in older adults associated gestational age with blood pressure, but not birth weight [54]**.** Apart from the factors mentioned above that potentially influence birth weight and the stress response, the following aspects might explain the lack of association between birth weight and the SNS-related stress response in our study: First, inter-individual variance between sAA baseline values is rather high, so analyzing a larger sample size (which is subject of discussion in the ‘Limitations’ section) might provide different results. Second, sAA as a correlate of SNS activity might actually not be associated with birth weight in female and male young adults. Therefore, future studies should evaluate both sAA and additional measures, such as heart rate or blood pressure, in a larger cohort to validate our findings.

We further assessed the relationship between birth weight and basal and reactivity values of IL-6. Basal IL-6, together with CRP, is used as a marker of chronic low-grade inflammation. In addition to the stress system, the inflammatory system is implicated in many psychiatric and somatic disorders, including PTSD [55]and CVD. Accordingly, IL-6 has predictive power for later life morbidity and mortality: raised levels of inflammatory markers, such as IL-6 and CRP, have been shown to predict the risk for future CVD[56] – which is also associated with birth weight [1]. Several studies focusing on the relationship between early life events and the immune system have found an association between prenatal adverse events and later inflammatory status [e.g., Ref. [19,57]. Research investigating the relationship between birth weight and systemic inflammation, mainly assessed by CRP levels, provides some evidence of an inverse association between birth weight and adult CRP concentrations [28]. However, results in children are conflicting [28], [58]], and a study examining different inflammatory markers and their relation to early life measures in an adult African population found no association between birth weight and those markers (including IL-6 and CRP) [59]. In our cohort, we also did not to detect a link between birth weight and baseline plasma IL-6 levels, nor did we find an association between birth weight and IL-6 reactivity: Acute psychological stress can induce an increase in plasma IL-6 concentrations [56]**,** and this reactivity has been shown to be elevated in individuals exposed to early life adversity [26]. The lack of any relationship between birth weight and IL-6 values in our cohort might be due to the fact that - similar to HPA axis activity - the inflammatory system can be influenced by a variety of postnatal factors, such as adverse exposures over the life course, genetic factors, or sleep quality [56]**.** We therefore conclude from our results that birth weight might not be a sufficiently sensitive marker to predict IL-6 values in adulthood, although we cannot exclude that investigating a larger cohort and/or a broader birth weight range could prove otherwise.

Later life events influencing the inflammatory and the stress system might also account for the lack of association between birth mode and any of the outcome variables investigated in our study. Delivery by Cesarean section has been linked to an increased disease risk in the child [60]**,** possibly mediated by a differential initial gut microbiome and a disrupted early priming of the stress- and immune system. Dinan and colleagues [61]have investigated the effect of delivery mode on the response of the HPA axis- and the inflammatory system to the TSST in a cohort of young male adults. Although participants born by C-section reported greater psychological stress and showed altered IL-1β and IL-10 levels, both their plasma IL-6- and their salivary cortisol responses to the TSST were comparable to those of vaginally delivered participants [61]. The results obtained from our cohort, with a majority of female participants, are in line with these findings. Since the main model was not significant, the statistical trend to an effect of birth mode on IL-6 reactivity was not evaluated here, but strongly suggests further analyses. Our present work extends the findings reported by Dinan et al. [61] with regard to SNS activity, assessed by sAA levels. As with the cortisol stress response, the mode of delivery did not predict basal sAA or sAA reactivity levels in our cohort.

We further tested whether the duration of breastfeeding predicts the biological markers assessed in our study. Breastfeeding is considered to have positive effects on a child's development [30] and to influence the development of HPA axis function in a number of ways. These include an improved quality of mother-infant interaction [62] and the breast milk itself, which also has an effect on the gut microbiome [30]**.** A study investigating the link between breastfeeding and cortisol stress response in 12-months-old infants showed that more weeks of breastfeeding predicted quicker cortisol recovery, whereas there was no association between breastfeeding and cortisol reactivity [62]. In our adult cohort, the duration of breastfeeding was not linked to baseline, nor to cortisol reactivity values, which is in line with results obtained from infants [62,63]. However, we saw a trend to a positive link between the duration of breastfeeding and cortisol recovery values. Although we calculated cortisol recovery from peak values, not in reference to pre-stress values, this trend matches the findings by Beijers et al. [62] and should be validated in future studies. Regarding the prediction of SNS activity, breastfeeding was not associated with sAA measures in our study. However, in infants, determining the impact of breastfeeding as potential confounding factor on sAA levels did not identify a significant association, either [63]. We neither detected a relation between breastfeeding and the inflammatory marker IL-6. This was unexpected, since breast milk provides immunological support to infants [30] and previous work has found an association between shorter durations of breastfeeding and elevated CRP levels in young adults [64] and adult women [65]. This suggests that investigating baseline CRP as biomarker of low-grade inflammation in the present context might be more reliable than IL-6, although assessing baseline IL-6 in a larger cohort might provide similar, significant results. However, regarding stress reactivity measures, it has to be considered that CRP levels do not seem to reliably respond to acute stress, in contrast to IL-6 [24]**.**

Finally, we tested whether the number of vaccinations (0–2 versus 3–7) received before the age of 4 months predicted any of our outcome measures. In the year 2000, children received up to 11 vaccines and 20 injections by the age of 2 years[29]. Evidence suggests that neonates are exceedingly capable of responding robustly and safely to most vaccines [66]**,** and early life vaccines clearly have a positive impact on later health by preventing diseases [67]. To the best of our knowledge, the relationship between early life vaccination status and markers of the stress and inflammatory system in adulthood, particularly in the context of psychosocial challenge, has not yet been investigated. Although the non-significant trend towards a relationship between higher baseline sAA levels and a higher number of early life vaccinations should be further investigated, the vaccination status at 4 months of age did not significantly predict basal or stress-related cortisol, sAA and IL-6 levels in our cohort. Since we have adjusted the threshold for dichotomization of the variable “vaccination status” to our sample characteristics, future studies with larger cohorts might increase the sensitivity of this variable. However, our current findings suggest that the number of vaccinations received in early life is not associated with changes in the stress- and inflammatory systems in adulthood.

### 6.1 Limitations

We have already mentioned some of the limitations that have to be considered when interpreting our results. First, our sample comprised a limited number of participants, the majority of whom were female. Applying various inclusion and exclusion criteria to minimize the impact of potential confounders, and maximize the quality of the data analyzed, further reduced our sample size. Future studies investigating larger cohorts with a more balanced sex distribution might reveal more subtle associations, and/or confirm the trend effects observed in the present study. Considering the impact of sex and sex hormone use on several of the parameters investigated here – as well as a focus on male subjects in the current literature – sex-specific analyses should be performed. In addition, future studies should account for non-responders, which was not done in this study due to the limited sample size, and for multiple testing. Second, our study was designed cross-sectionally. As discussed above, developmental stages and events later in life, such as pubertal stress recalibration [68], or periods of elevated stress, may influence the stress- and inflammatory response. A longitudinal study design with repeated testing may therefore provide more comprehensive data. This also applies to the assessment of additional markers of the HPA axis, SNS and inflammatory system. Third, there are additional sources of intra- and inter-individual variability in stress response levels, such as genetic and epigenetic factors, lifestyle and psychological variables [for review see Refs. [31]**]**, which we could not account for in our study. Fourth, we cannot exclude a certain degree of imprecision in the birth weight records and the retrospectively assessed breastfeeding duration. Nonetheless, the perinatal markers analyzed in this study are considered to be more objective and quantifiable than retrospective reports of prenatal adverse events.

## 7 Conclusions

The results of the present study confirm an association between birth weight and the salivary cortisol response to psychosocial stress in a cohort of male and female young adults recruited from the general population, extending previous research. Still, birth weight is a crude marker of prenatal conditions, which might explain why it was not related to sAA and IL-6 outcomes in our study. Future studies should therefore investigate the association between birth weight and sAA/IL-6 in subjects who were exposed to defined prenatal adverse events. Furthermore, our results indicate that neither the birth mode, nor the duration of breastfeeding, nor the vaccination status at 4 months of age have a long-term impact on the markers of the stress- and inflammatory system assessed here. The results of our exploratory study should be confirmed in larger cohorts and with additional biomarkers.

## Ethics approval and consent to participate

The study was approved by the Local Ethics Committee of the Friedrich-Alexander University Erlangen-Nuremberg and was conducted in accordance with the Declaration of Helsinki. All participants provided written informed consent.

## Consent for publication

Not applicable.

## Availability of data and materials

The datasets used and/or analyzed during the current study are available from the corresponding author on reasonable request.

## Funding

This research did not receive any specific grant from funding agencies in the public, commercial, or not-for-profit sectors.

## CRediT authorship contribution statement

**Anne-Christine Plank:** Writing – original draft, Formal analysis, Data curation. **Janina Maschke:** Writing – review & editing, Investigation, Data curation. **Stefan Mestermann:** Writing – review & editing. **Johanna Janson-Schmitt:** Writing – review & editing, Investigation, Data curation. **Sarah Sturmbauer:** Writing – review & editing, Investigation, Data curation. **Anna Eichler:** Writing – review & editing, Formal analysis, Conceptualization. **Nicolas Rohleder:** Writing – review & editing, Supervision, Investigation, Conceptualization.

## Declaration of competing interest

The authors declare that they have no known competing financial interests or personal relationships that could have appeared to influence the work reported in this paper.
